# Serum Protein Dynamics in Early Pregnancy Loss Management: Findings from the PreFaiR Trial

**DOI:** 10.21203/rs.3.rs-7577064/v1

**Published:** 2025-09-25

**Authors:** Jagyashila Das, Sarah M. Gutman, Arden McAllister, Mary Sammel, Courtney A. Schreiber, Shefali Setia Verma

**Affiliations:** University of Pennsylvania Perelman School of Medicine; University of Pennsylvania Perelman School of Medicine; University of Pennsylvania Perelman School of Medicine; University of Colorado Anschutz Medical Campus, Colorado School of Public Health; University of Pennsylvania Perelman School of Medicine; University of Pennsylvania Perelman School of Medicine

**Keywords:** early pregnancy loss, miscarriage, mifepristone, misoprostol, proteomics, biomarkers, medical management, treatment prediction

## Abstract

Medical management of early pregnancy loss (EPL) achieves ~90% efficacy with mifepristone-misoprostol combination versus ~70% with misoprostol alone, yet biomarkers predicting individual treatment response remain unknown. We performed proteomic analysis of serum samples from 59 participants in the PreFaiR trial, comparing pre- and post-treatment protein expression between responders and non-responders. Combination therapy showed 5 baseline predictors (LAP-TGF-β1, IL17RB, MYOC, CDH1, CD97) with higher expression in responders, while misoprostol monotherapy demonstrated 10 predictors with bidirectional patterns. Treatment-induced changes differed markedly: combination therapy showed significant alterations in 4 proteins, while misoprostol triggered changes in 30 proteins. TNNI3 emerged as a molecular efficiency marker, which showed almost no change between pre- and post-treatment levels in successful combination therapy but substantially declining post-treatment with misoprostol monotherapy. DPP7 showed opposite regulation between treatments among responders. These distinct molecular signatures provide evidence for treatment-specific mechanisms and offer potential biomarkers for personalized EPL management.

## INTRODUCTION

Early pregnancy loss (EPL), also referred to as miscarriage, is common, occurring in up to 25% of pregnancies^1^. Following a diagnosis of EPL, patients may opt for expectant management (waiting for the pregnancy tissue to pass without intervention), medical management (taking medications to expedite uterine emptying), or procedural management through uterine aspiration^2^. Many patients who wish to avoid a procedure choose medical management due to the increased control over the timing of bleeding and cramping that accompanies tissue expulsion^3,4^.

The landscape of medical management for EPL has evolved significantly in recent years^5^. While misoprostol alone has been historically used, the introduction of combination therapy with mifepristone pretreatment has markedly improved treatment efficacy, and, by expediting the tissue expulsion process, can reduce the incidence of adverse outcomes resulting from EPL^6,7^. Mifepristone is a 19-nor steroid that acts as competitive progesterone-receptor antagonist and glucocorticoid-receptor antagonist, priming the myometrium and cervix for prostaglandin activity^8,9^. The superiority of combination therapy was definitively demonstrated in a 2018 randomized controlled trial, which led to the establishment of mifepristone (200 mg oral) followed by misoprostol (800 mcg vaginal) as the standard of care for EPL medical management in the United States^10^. Misoprostol alone continues to be used when mifepristone is not available, but the efficacy of the single-drug regimen is low, especially when the cervical os (the external opening in the cervix of the endocervical canal) is closed, and patients may need to use several doses^11^.

While the adoption of combination treatment with mifepristone has boosted the efficacy of EPL medical management to approximately 90%, the emotional and physical burden of treatment failure remains significant^12^. Identifying reliable predictors of success or failure, both before treatment to aid with clinical decision-making, and after treatment, to aid in confirmation of treatment success, would empower patients to make more informed decisions^10^. We examined pre- and post-treatment changes in serum protein expression in a cohort of women who received either misoprostol alone or combination misoprostol with mifepristone in the Comparative Effectiveness of Pregnancy Failure Management Regimens (PreFaiR) trial^9^.

In this manuscript we aim to identify proteomic signatures that would enable personalized treatment selection, as well as biomarkers to predict individual treatment response. We investigated protein expression changes between responders and non-responders, as well as between patients receiving misoprostol alone versus the combination regimen. We used multi-modal analytical framework to investigate the molecular underpinnings of EPL treatment response.

## MATERIALS AND METHODS

This was a planned secondary analysis of a randomized controlled trial, the Comparative Effectiveness of Pregnancy Failure Management Regimens (PreFaiR)trial, which evaluated the clinical efficacy and safety of mifepristone pretreatment compared to misoprostol alone for the medical management of early pregnancy loss.^3^ The trial was approved by the institutional review boards (IRB) at the University of Pennsylvania, the University of California, Davis, and the Albert Einstein College of Medicine (ethics approval number: 818434), and full protocol and trial results have been previously published.^9^ This study was conducted according to US and international standards of Good Clinical Practice (FDA Title 21 part 312 and International Conference on Harmonization guidelines), applicable government regulations and Institutional research policies and procedures. As a part of the study protocol, participant blood samples were collected before and after medication administration.

### Study Design, Participants and specimen collection

After confirming patient eligibility and obtaining informed consent, blood samples were collected in BD Vacutainer rapid serum tubes at pretreatment (day of randomization, pretreatment) and at a post-treatment visit that occurred 4 days after treatment initiation. This analysis focused exclusively on missed miscarriages with closed cervical os to ensure clinical homogeneity. Participants were matched within 7 days of gestational age to minimize confounding by pregnancy duration. After collection, the tubes were gently inverted 5 times and the sample allowed to clot at room temperature for up to 2 hours prior to centrifugation. After centrifugation, serum samples were transferred to labeled cryovials using a sterile pipette, then flash frozen in liquid nitrogen and stored in a −80°C freezer.

For this proteomic analysis, we included a subset of non-responders and matched responders. Participants in each of the study arms (pretreatment with mifepristone followed by misoprostol or misoprostol alone) were classified as either “treatment failure” (non-responders) or “treatment success” (responders); treatment success was defined as expulsion of the gestational sac by the first post-treatment visit with 1 dose of misoprostol and no additional surgical or medical intervention within 30 days after treatment. Responder and non-responder sample pairs (collected at pretreatment and post-treatment) for each treatment arm were matched within 7 days of gestation for inclusion in this analysis. We included two treatment groups in the analysis. The misoprostol alone group consisted of 29 individuals who received only misoprostol, while the pretreatment group included 30 individuals who received mifepristone pretreatment before misoprostol. Each group had a nearly equal distribution of individuals with successful and unsuccessful outcomes, serving as non-responders and responders, respectively.

### Proteomic analysis

Targeted proteomic analysis was conducted by a commercial proteomics company (Olink Proteomics, Uppsala, Sweden) using a technology called “Proximity Extension Assay,” which uses minimal amounts of serum or plasma to conduct multiple immunoassays of proteins. In this analysis, we evaluated the expression of a total of 1009 proteins in the pre- and post-treatment sample pairs from each matched non-responders and responders. Protein expression levels were measured pre- and post-treatment. We calculated the fold change in protein expression for each protein by comparing post-treatment to pre-treatment levels.

A total of 1009 proteins were assayed. The protein expression values were median normalized prior to statistical analysis^13^. The distribution of protein expression before and after normalization is presented in Figure S1. To prioritize proteins with meaningful expression variability, we selected those with variance exceeding the proteome-wide median threshold (>0.177). This filtering step reduced the dataset to 512 proteins exhibiting substantial variation across samples.

### Statistical Analysis

To identify pre-treatment proteins predicting treatment success, we compared baseline protein levels between responders and non-responders using Mann-Whitney U test. We performed separate analyses for each treatment group, recognizing that distinct drug mechanisms may yield different predictive biomarkers.

Furthermore, we calculated change scores (Δ = post-treatment - pre-treatment) for each protein for all patient participants. These change scores were then compared between responders and non-responders using Mann-Whitney U tests within each treatment group.

To assess differential molecular mechanisms among responders, we performed the same test to compare protein change patterns across treatment arms..

For effect size estimation we computed the rank biserial correlation (r) for concluding significant (p < 0.05) clinically meaningful differences. We prioritized proteins demonstrating large effect sizes (|r| ≥ 0.5).

### Multi-modal proteomic feature selection framework

We employed two complementary feature selection approaches to identify proteins with non-linear relationships and interaction effects that standard univariate tests might miss. Mutual information (MI) analysis was performed using the *infotheo* package to quantify the predictive power of each protein without assuming linear relationships between protein levels and treatment outcomes^14^. Random Forest (RF) importance scores were derived using the *randomForest* package with 100 trees to identify proteins that contributed most strongly to outcome prediction while accounting for potential protein-protein interaction effects^15^. Mean Decrease in Gini index was used as the importance metric. All statistical analyses were performed using R software, version 4.3.2^16^. All data wrangling performed using R tools like *tidyverse, dplyr*, plots were generated using *ggplot2*^17,18,19^.

## RESULTS

### Participant Characteristics

#### Demographics

A total of 59 participants with non-missing data were included in the analysis, with almost equal distribution between the combined (mifepristone and misoprostol) group (n=30) and the misoprostol-only group (n=29). Both groups had similar age distributions, with a median age of 30 years in both groups (range: 18–42 years in the pretreatment group; 18–40 years in the misoprostol-only group), summarized in Table 1.

#### Obstetric History

Baseline obstetric characteristics showed both groups had predominantly parous participants with prior pregnancy experience. Among those experiencing their first pregnancy, 7 participants were in the combined group and 4 in the misoprostol group. The majority of participants in both groups were parous (57% in combined23; 79% in misoprostol-only group), with a median of 2 previous pregnancies in both groups. Both groups had similar median gestational ages at enrollment (matched within 7 days) and comparable proportions of treatment success (46.7% vs 44.8%), indicating balanced groups for our primary analyses.

#### Race/Ethnicity

Race distribution showed some differences between groups based on the available electronic health record (EHR) data. Majority (50%) of the subjects in combined group self-identified as white, compared to 27.6% in the misoprostol only group; whereas there were more Black/African American participants in the misoprostol-only group (44.8%) compared to the combined pretreatment group (36.7%). Participants that self-identify as Asians appeared more frequently in the miso group (10.3%) than in the combined group (3.3%). Race was categorized as Unknown for 10% of participants in the combined pretreatment group and 17.2% in the misoprostol-only group.

#### Proteomic signatures distinguish treatment responders from non-responders

Our multi-faceted approach examined baseline predictors, treatment-induced changes, and differential patterns between treatment regimens.

##### a. Baseline protein levels predict treatment response

**Figure 1:**
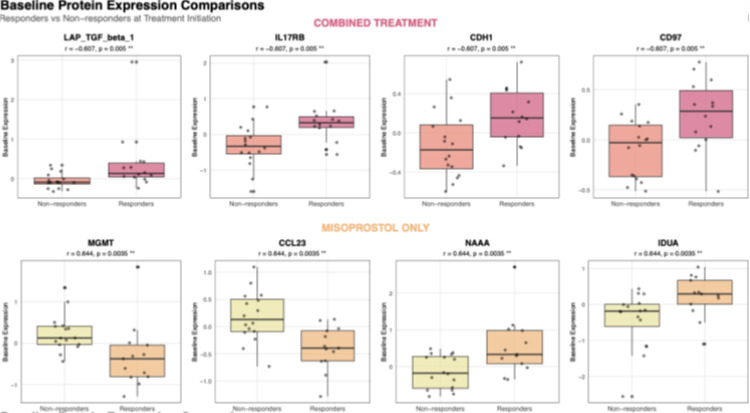
Boxplot showing the most differential protein expression (baseline vs post-treatment) between drug groups. Each outlined box represents specific proteins, top row shows 4 of the most differentially expressed proteins between non-responders and responders prior to treatment (baseline levels) with mifepristone followed by misoprostol (combined). Bottom row represents 4 most significantly differentially expressed proteins prior to misoprostol-only treatment, between non-responders and responders. Salmon and yellow from top and bottom rows respectively denotes non-responders, whereas pink and orange denotes responders from each drug group respectively.

Analysis of pre-treatment protein levels from both combined and misoprostol-only treatment groups identified distinct signatures between future responders and non-responders, with treatment-specific patterns emerging for each regimen (Table 2, Figure 1). Positive rank biserial R values mean higher expression in post-treatment samples; negative values indicate higher protein expression levels in pretreatment.

In the combined mifepristone-misoprostol group, 5 proteins showed large effect (|r| > 0.5) with significant differences (p<0.05) between future responders and non-responders. The strongest baseline predictors for combined treatment included LAP TGF beta1 (Latency-Associated Peptide transforming growth factor-beta 1) (r=−0.61, p=0.005), IL17RB (r=−0.59, p=0.006), and MYOC (r=−0.53, p=0.015), along with CDH1 (cadherin 1) and CD97 (Cluster of Differentiation 97, also known as ADGRE5, adhesion G protein-coupled receptor E5), all showing higher baseline protein expression in responders.

The misoprostol monotherapy group exhibited more pronounced baseline differences, with 10 proteins showing large effect sizes (|r| > 0.5), and significant differences (p < 0.05).

The top predictors for misoprostol-only group included IDUA (alpha-L-iduronidase) and NAAA (N-acylethanolamine acid amidase) (p<0.05, |r|> 0.5) with higher baseline levels predicting success. COL18A1 (Collagen type XVIII alpha 1 chain), PRKCQ (Protein Kinase C Theta), CDH1, CD8 ((Cluster of Differentiation 8), REG1A (regenerating family member 1 alpha), CCL14 (C-C motif chemokine ligand 14), MGMT (O-6-methylguanine-DNA methyltransferase) all with nomically significant p value (p<0.05) showed lower baseline protein levels predicting treatment success.

##### b. Treatment-induced protein changes associated with clinical outcomes

**Figure 2:**
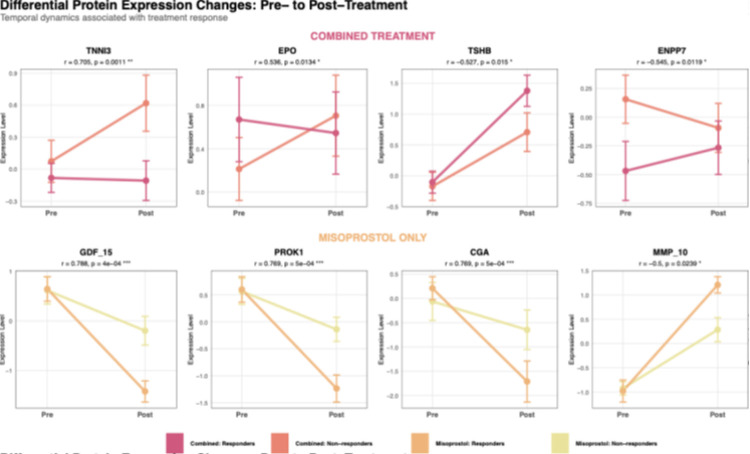
Line plot showing change in baseline vs post-treatment protein expression between drug groups. Each outlined box represents specific proteins, upper row shows 4 of the most differentially expressed proteins between non-responders and responders before and after treatment with mifepristone followed by misoprostol (combined). Bottom row represents 4 most significantly differentially expressed proteins between non-responders and responders before and after treatment with misoprostol only. Salmon and yellow from top and bottom rows respectively denotes non-responders, whereas pink and orange denotes responders from each drug group respectively.

Differential protein expression changes between pre- and post-treatment timepoints revealed distinct patterns associated with treatment success (Table 3, Figure 2). Positive rank biserial R values mean the pretreatment protein levels are higher in non-responders compared to that of responders, negative values mean the protein levels higher in responders between pre- and post-treatment. In the combined treatment group, 4 proteins showed change in patterns between responders and non-responders exhibiting large effect sizes (p<0.05, |r| >0.5). The misoprostol monotherapy group demonstrated 30 proteins with large differential effects (p<0.05, |r| >0.5). Individuals responding to combination therapy showed greater decreases in TNNI3 (r = 0.71, p = 0.001).

##### c. Responders display treatment-specific proteomic patterns

Among patients with successful outcomes, direct comparison of protein changes between treatment regimens revealed 15 proteins with significantly different patterns (p<0.05), with effect sizes |r| > 0.5 (Table 4). Positive rank biserial R values mean the misoprostol-only display more change in expression compared to that of combined drug, negative mean the protein level change observed in combined therapy is higher than Misoprostol only group.

DPP7 emerged as the most differentially regulated protein (|r|=0.75, p<0.001), increasing in combined treatment responders while decreasing in misoprostol responders.

**Figure 3:**
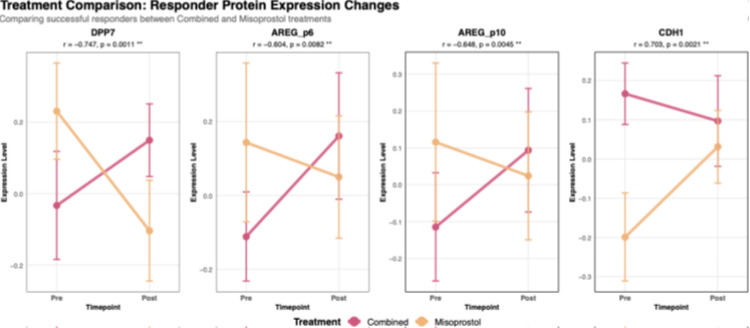
Line plot showing pre- and post-treatment change in protein expression between responders from the two drug groups. The lines show opposing protein-expression trends in responders only, in baseline and post treatment levels. Each outlined box represents specific proteins. Pink and orange denote responders from combined and misoprostol-only drug groups respectively.

Both AREG peptides (p6 and p10) displayed similar inverse relationships between the treatment groups. Both peptides showed upward trajectory with combined therapy, contrasting with downward trajectory for misoprostol only treatment in successful cases (Figure 3). The consistent patterns across both AREG peptides suggest coordinated regulation of AREG expression that differs fundamentally between treatment modalities in successful cases.

#### Protein importance and module classification

**Figure 4:**
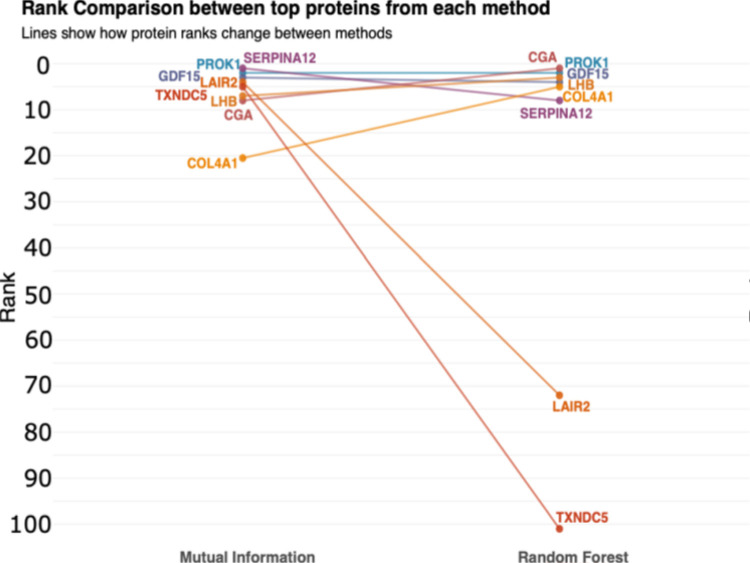
Protein ranking concordance between mutual information and random forest feature selection methods. Slope graph showing rank changes for the top proteins from each method. Lines connect the same protein’s rank between methods, with consistent proteins shown in blue/purple, and method-specific proteins in orange.

To better capture potential non-linear relationships and interaction effects that univariate tests might miss, we implemented two complementary methods (Mutual Information, and Random Forest) to identify the proteins with the strongest association with treatment outcomes effectively captured proteins with significant predictive value for treatment success. Across all three methods, four proteins consistently emerged as most significant: PROK1, GDF15, TSHB, and SIGLEC6 (Figure 4). PROK1, GDF15, SERPINA12, LHB, CGA and COL4A1 show high concordance in both methods. The convergence of PROK1, GDF15, and CGA across three distinct analytical approaches—treatment-induced changes, Mutual Information, and Random Forest—represents a particularly robust finding.

On contrary, LAIR2, and TXNDC5 show substantial rank differences, indicating method-specific importance. These results demonstrate that our multi-modal approach identified a core set of proteins with consistent predictive value across different analytical frameworks, while also identifying proteins that lead to specific interaction patterns within the proteomic landscape.

## DISCUSSION

Medical management of EPL provides patients with a safe, effective treatment when procedures may be inaccessible or undesirable. This comprehensive proteomic analysis reveals distinct molecular signatures predicting treatment response in EPL management, with marked differences between combination therapy and misoprostol monotherapy in both baseline predictors and treatment-induced changes^20^. The combination of multi-modal approaches addresses the critical blind spots inherent to each method when used in isolation, especially with high dimensional proteomic dataset with limited sample size. Our multi-modal analytical framework combines univariate testing with machine learning approaches (Random Forest) and information theory (Mutual Information) to capture both individual protein effects and complex protein-protein interactions, as well as non-linear relationships that may be critical in high-dimensional proteomic datasets with limited sample sizes.

Individuals with higher protein expression in pretreatment (baseline) levels of latency-associated peptide TGF-β1, CDH1, IL17RB, CD97, and MYOC given combined mifepristone-misoprostol therapy predicts successful outcome. Transforming growth factor-β (TGF-β1) is a cytokine which maintains immune homeostasis during pregnancy, thereby playing crucial role in pregnancy outcome. Higher levels of TGF-β1 during pregnancy leads to recurrent pregnancy loss, preeclampsia (PE)^21^. On contrary, significant lower expression of CD97 has also been previously reported to be associated with PE^22^. Circulating levels of Interleukin 17 receptor B (IL17RB) has also been found to be pivotal in maintaining and resulting in successful pregnancy outcome. While elevated levels are beneficial for a healthy pregnancy, excessive levels are associated with pregnancy related complications^23^. IL17 has previously been used as a biomarker for PE and recurrent pregnancy loss (RPL)^24^. Evaluation of IL17RB protein levels before treatment initiation may represent a valuable strategy for predicting response to combined therapy, underscoring the need for further clinical investigation. In contrast, the role of MYOC in pregnancy remains unclear, and further studies are needed to elucidate its potential contribution to treatment response or pregnancy-related pathways.

Comparative analyses reveal differential expression patterns before and after combined therapy, in 4 proteins (TNNI3, EPO, TSHB, and ENPP7), and a different set of 30 proteins in individuals given misoprostol-only treatment. A steep declining expression of GDF15 levels from before to after treatment in responders from misoprostol-only treatment group, suggests putative role involved in successful response to misoprostol-only treatment. GDF15 is known for its role in inflammation and tissue repair, which may reflect the physiological response to the administered drugs^25,26^. GDF15 is a circulating member of the TGF-ß superfamily; it has been previously reported as a key player in the pathogenesis of nausea and vomiting of pregnancy (NVP) or hyperemesis gravidarum (HG)^27,28^. Similarly, PROK1 also showed declining trend from pre- and post-treatment in the misoprostol-only treatment group. PROK1 protein is expressed in multiple tissues including the ovaries, decidua and placenta^29^. It has been shown to play key role in vascularization and implantation^30^. Decreased PROK1 expression has also been associated with preeclampsia and recurrent miscarriage^31,32^. Additionally, previous reports suggests TSHB as a crucial protein for maintenance and successful reproductive health, essentially, dysregulation of the protein leads to infertility, miscarriage, as well thyroid disturbances during pregnancy, or even after parturition^33,34^. Responders to combined therapy exhibited higher TSHB expression, suggesting that lower pre-treatment TSHB levels could serve as a candidate biomarker for predicting treatment success and merit further study.

The convergence of PROK1, GDF15, TSHB, and SIGLEC6 across all three methods - strongly supports their relevance as putative biomarkers of treatment response, for future validation. Furthermore, CGA (Glycoprotein Hormone Alpha Subunit) came up as significant and consistent in both univariate (test to compare between responders and non-responders given Misoprostol-only treatment) and multi-modal feature selection methods(MI and RF), might potentially indicate its role as the hormonal co-ordinator, given its role as the alpha subunit of hCG, TSH, FSH, and LH—its decline in responders indicate broad hormonal shutdown. CGA changes reflect multiple endocrine axes, for example, the reproductive placental axis through hCG, the thyroid axis through TSH, the gonadotropin-ovarian axis through follicle stimulating hormone (FSH) and Luteal hormone (LH), simultaneously^35,36^.

In the pretreatment regimen, DPP7 expression increased significantly in responders compared to non-responders. Conversely, in the misoprostol-only group, DPP7 expression decreased regardless of treatment outcome. This specific upregulation of DPP7 in successful pretreatment participants, together with the minimal protein expression changes observed in the misoprostol-only group, suggests that the pretreatment activates distinct molecular pathways that may enhance treatment efficacy.

Our analysis of drug-specific comparison effects identified another distinct protein signature specifically associated with the pretreatment regimen. The differential expression of amphiregulin (AREG) isoforms in non-responders versus responders provides insight into potential mechanisms of drug resistance. AREG (p6 and p10) showed differing trend in expression between misoprostol (declining) and combined group (increasing). AREG is a known regulator of placental function, with increased expression in human amniotic fluid^37,38^.

TNNI3 (Cardiac troponin I 3), typically associated with cardiac and smooth muscle contraction, showed a particularly informative pattern across our analyses^39^. This specific protein demonstrates treatment-specific patterns. In the combined treatment group, responders showed almost no change in TNNI3 expression levels as compared to non-responders, who showed marked increase post-treatment (r = 0.71, p = 0.001). Notably, when comparing successful outcomes between treatment arms, TNNI3 levels were found to decrease more substantially in the combination therapy than in misoprostol monotherapy (r = 0.52, p = 0.024).

Several limitations inherent to secondary analyses of clinical trial data should be considered when interpreting these results. A primary constraint is the absence of a comparison group comprising individuals who experienced spontaneous abortion, which would have provided valuable baseline data for contrasting with medication-induced outcomes. Without a placebo control, we cannot distinguish drug-induced protein changes from those associated with the miscarriage process itself. Our analyses identify biomarkers associated with successful treatment completion rather than drug response per se.

However, these markers retain clinical utility for treatment selection and monitoring. Additionally, as this investigation was conducted as a sub-study within a larger clinical trial, due to the low number of failures in the trial overall, and our sample size was relatively small, which may limit the generalizability of our findings. As such, these findings should be considered exploratory and can be used for follow-up studies. Despite these limitations, our preliminary data show that proteomic biomarkers of EPL treatment success may have potential applications both before and after intervention. Pre-treatment biomarkers identified in this study should be further validated, which could thereby serve as decision-making tools to guide therapeutic approaches, while post-treatment, they offer potential for non-invasive confirmation of successful outcomes. This research demonstrates that proteomic approaches hold considerable promise for biomarker discovery in this clinical context. These identified biomarkers not only help elucidate the underlying molecular mechanisms of early pregnancy failure but also provide a foundation for developing predictive models. Such models could ultimately help patients experiencing EPL access the most effective treatment options and provide reliable confirmation of treatment success without invasive procedures.

## CONCLUSIONS

Our exploratory proteomic analysis has identified distinct protein expression signatures associated with successful medical management of early pregnancy loss, comparing outcomes between misoprostol monotherapy and pretreatment with misoprostol. Our findings reveal distinct protein signatures to help with clinical decision-making at the time of treatment option consideration, as a noninvasive approach to assessing treatment success, as well as understand the mechanistic underpinnings. The current study reveal distinct protein signatures that predict treatment success as well as illuminate the differential mechanisms of action between mifepristone-misoprostol combination therapy and misoprostol monotherapy on EPL. With further validation in larger cohorts, these proteomic signatures have the potential to transform clinical practice by enabling personalized treatment selection, improving patient counseling, and providing non-invasive methods to confirm treatment success—ultimately advancing care for patients experiencing early pregnancy loss.

## Supplementary Material

Supplementary Files

This is a list of supplementary files associated with this preprint. Click to download.

- SupplementaryFigurePrefairOlink.docx

## Figures and Tables

**Table 1:** Table summarizing the participant demographic information

Characteristic	Combined (n=30)	Misoprostol only (n=29)
**First pregnancy**, n (%)		
First pregnancy	7 (23.3)	4 (13.8)
Not first pregnancy	23 (76.7)	25 (86.2)
**Parity**, n (%)		
Nulliparous (parity = 0)	13 (43.3)	6 (20.7)
Parous (parity = 1)	17 (56.7)	23 (79.3)
**Race distribution 2**, n (%)		
Black/African American	11 (36.7)	13 (44.8)
White	15 (50.0)	8 (27.6)
Asian	1 (3.3)	3 (10.3)
Other/Unknown	3 (10.0)	5 (17.2)
**Treatment outcome**, n (%)		
Success	14 (46.7)	13 (44.8)
Failure	16 (53.3)	16 (55.2)

**Table 2:** List of proteins showing significant difference in baseline (pretreatment) levels, which varied significantly between the two types of drugs administered.

Drug	protein	p-value	rank biserial R
Combined	LAP TGF beta1	0.005	−0.607
	IL17RB	0.006	−0.589
	MYOC	0.015	−0.527
	CDH1	0.021	−0.500
	CD97	0.021	−0.500
Misoprostol only	MGMT	0.004	0.644
	MGMT	0.004	0.635
	CCL14	0.005	0.625
	REG1A	0.007	0.596
	CDH1	0.017	0.529
	CD8A	0.017	0.529
	PRKCQ	0.020	0.505
	COL18A1	0.024	0.500
	NAAA	0.017	−0.529
	IDUA	0.012	−0.558

**Table 3:** List of proteins showing significant differences (p< 0.05, |r| > 0.5) in changing patterns between responders and non-responders, in the two types of drugs administered.

Drug	Protein	p-value	rank biserial R
Combined	TNNI3	0.001	0.705
	EPO	0.013	0.536
	TSHB	0.015	−0.527
	ENPP7	0.012	−0.545
Misoprostol only	GDF15	0.000	0.788
	PROK1	0.000	0.769
	CGA	0.000	0.769
	PAPPA	0.001	0.731
	ANGPT2	0.002	0.683
	NAAA	0.002	0.683
	TFPI2	0.002	0.683
	TRAP	0.005	0.625
	EpCAM	0.005	0.625
	SIGLEC6	0.005	0.625
	IGSF3	0.006	0.606
	REN	0.008	0.587
	APN	0.013	0.548
	PON3	0.013	0.548
	ARSB	0.013	0.548
	SELP	0.015	0.538
	IGFBP1	0.015	0.538
	EGFR	0.015	0.538
	PDGF subunit A	0.015	0.538
	ALCAM	0.017	0.529
	BLM hydrolase	0.017	0.529
	CCL24	0.017	0.529
	PAI	0.019	0.519
	OMG	0.019	0.519
	ST2	0.021	0.510
	PECAM1	0.021	0.510
	SMOC2	0.021	0.510
	IL27	0.024	0.500
	MANF	0.024	0.500
	MMP10	0.024	−0.500

**Table 4:** List of proteins showing significant differential expression patterns in responders from combined and misoprostol-only treatment groups.

protein	p-value	rank_biserial r
DPP7	0.001	−0.747
AREG_p10	0.005	−0.648
AREG_p6	0.008	−0.604
BLM_hydrolase	0.014	−0.560
CLSTN2	0.014	−0.560
ARSB	0.016	−0.549
CD164	0.016	−0.549
TR_AP	0.019	−0.538
SERPINB6	0.021	−0.527
SDC4	0.021	−0.527
NOMO1	0.024	−0.516
SOST	0.024	−0.516
TNNI3	0.024	0.516
PAM	0.011	0.582
CDH1	0.002	0.703

## Data Availability

The proteomic datasets generated during this study will be deposited in https://figshare.com/ upon manuscript acceptance. Due to patient privacy requirements and IRB restrictions, clinical metadata will be available upon reasonable request to the corresponding author with appropriate data use agreements. The PreFaiR trial is registered at ClinicalTrials.gov (NCT02012491).
